# Development of a Convenient *In Vivo* Hepatotoxin Assay Using a Transgenic Zebrafish Line with Liver-Specific DsRed Expression

**DOI:** 10.1371/journal.pone.0091874

**Published:** 2014-03-13

**Authors:** Xiaoyan Zhang, Caixia Li, Zhiyuan Gong

**Affiliations:** Department of Biological Sciences, National University of Singapore, Singapore, Singapore; Institute of Cellular and Organismic Biology, Taiwan

## Abstract

Previously we have developed a transgenic zebrafish line (LiPan) with liver-specific red fluorescent protein (DsRed) expression under the *fabp10a* promoter. Since red fluorescence in the liver greatly facilitates the observation of liver in live LiPan fry, we envision that the LiPan zebrafish may provide a useful tool in analyses of hepatotoxicity based on changes of liver red fluorescence intensity and size. In this study, we first tested four well-established hepatotoxins (acetaminophen, aspirin, isoniazid and phenylbutazone) in LiPan fry and demonstrated that these hepatotoxins could significantly reduce both liver red fluorescence and liver size in a dosage-dependent manner, thus the two measurable parameters could be used as indicators of hepatotoxicity. We then tested the LiPan fry with nine other chemicals including environmental toxicants and human drugs. Three (mefenamic acid, lindane, and arsenate) behave like hepatotoxins in reduction of liver red fluorescence, while three others (17β-estradiol, TCDD [2,3,7,8-tetrachlorodibenzo-*p*-dioxin] and NDMA [N-nitrosodimethylamine]) caused increase of liver red fluorescence and the liver size. Ethanol and two other chemicals, amoxicillin (antibiotics) and chlorphenamine (pain killer) did not resulted in significant changes of liver red fluorescence and liver size. By quantitative RT-PCR analysis, we found that the changes of red fluorescence intensity caused by different chemicals correlated to the changes of endogenous *fabp10a* RNA expression, indicating that the measured hepatotoxicity was related to fatty acid transportation and metabolism. Finally we tested a mixture of four hepatotoxins and observed a significant reduction of red fluorescence in the liver at concentrations below the lowest effective concentrations of individual hepatotoxins, suggesting that the transgenic zebrafish assay is capable of reporting compound hepatotoxicity effect from chemical mixtures. Thus, the LiPan transgenic fry provide a rapid and convenient in vivo hepatotoxicity assay that should be applicable to high-throughput hepatotoxicity test in drug screening as well as in biomonitoring environmental toxicants.

## Introduction

Hepatotoxins are toxic chemicals that damage the liver or cause liver functional disorders. Some hepatotoxins are relevant to environmental contamination while others may be related to pharmaceutical uses. Hepatotoxins have a variety of mechanisms causing liver damages leading to different grades of liver conditions such as zonal necrosis, hepatitis, cholestasis, steatosis, granuloma and even neoplasm [1], [2]. As the liver is the most important organ in drug metabolism, one of the major causes of drug attrition during the pre-approval and post-approval stages of the drug development and marketing process is drug-induced liver injury [3]. Thus, it is crucial and important to develop rapid and convenient approaches not only for monitoring environmental contaminants but also for detection of potential hepatotoxicity in drug candidates screening.

To test hepatotoxicity of chemical compounds, a variety of assays have been developed. For *in vitro* study, human HepG2 cells have been popularly used with a capability of developing to a high content screening assay for screening a broad range of compounds [4] and the assay could be further improved by enhanced expression of major P450 enzymes [5]. There are also pluripotent stem cell-derived hepatocyte-like cells to serve as an additional method of detecting hepatotoxicity in the drug development progress [6]. In addition to measurement of conventional liver enzyme activities in blood such as GGT (Gamma-glutamyltransferase), ALT (alanine aminotransferase) and AST (aspartate aminotransferase), which are released to blood due to liver damage [7], now different types of hepatotoxins could be distinguished by screening different biomarkers; for example, a PCR array based assay is available to analyze the expression of 84 key genes implicated as potential biomarkers of liver toxicity [8].

Although the in vitro assays for hepatotoxicity has been well established, the hepatotoxicity test in *in vivo* animal models such as in mice is generally costly and requires a long time to complete. In contrast, the zebrafish (*Danio rerio*) is now an increasingly popular vertebrate model in biological research [9]). As an experimental model, the zebrafish has high fecundity with daily availability of hundreds of eggs in each spawning and transparent embryos to facilitate analyses of embryonic development, and requires less space and low cost in husbandry. Compared with in vitro cell culture systems, the zebrafish is an in vivo model and provides valuable information in a physiological context. In recent years, zebrafish embryos have also been applied as a predictive model for assessing drug-induced toxicity, including cardiotoxicity, hepatotoxicity, neutotoxicity and developmental toxicity assessment [10]–[12].

The LiPan [*Tg(fabp10a:DsRed;elaA:egfp*)] transgenic zebrafish we previously generated for developmental analyses have constitutive expression of DsRed fluorescent protein in the liver and green fluorescent protein (GFP) in the exocrine pancreas [13]. Since the red fluorescence in the liver makes the observation of liver in live fry easily, we envisioned that this transgenic line might provide a useful tool in analyses of hepatotoxicity as changes of liver fluorescent intensity and size in the LiPan fry could be easily and quantitatively analyzed. In this study, we first tested four well established hepatotoxins (acetaminophen, aspirin, isoniazid and phenylbutazone) in LiPan fry. Acetaminophen is a well-known hepatotoxin that induces hepatic damages, oxidative stress and cellular necrosis [14]. Aspirin is a pleiotropic drug and has been reported to be a dose-related intrinsic hepatotoxin [15]. Isoniazid is known to cause oxidative stress and apoptosis in the liver [16], [17]. Phenylbutazone is a poisonous toxin as well as a hepatotoxin with severe oxidative stress in the liver [18]. Indeed we observed dosage-dependent decreases of both red fluorescence intensity in the liver and the size of the liver by treatment with the four established hepatotoxins. Then we further tested nine other chemicals including both environmental toxicants and human drugs. While some chemicals caused similar reductions of liver red fluorescence and liver size, other chemicals either increased the liver red fluorescence and liver size or had no effect. Our data indicate that the LiPan transgenic fry provide a rapid and convenient hepatotoxicity assay that should be applicable to high throughput hepatotoxicity test in drug screening as well as in biomonitoring of environmental toxicants.

## Materials and Methods

### Ethics Statement

All experimental protocols were approved by Institutional Animal Care and Use Committee (IACUC) of National University of Singapore (protocol 079/07).

### Materials

The LiPan transgenic zebrafish line Tg(lfabp10a: DsRed; elaA:EGFP) was established previously in our laboratory [13]. 13 chemicals tested in the present study were purchased from different commercial sources: acetaminophen (Sigma, A7085), ethanol (Merck, 1.00983.2500), lindane/hexachlorocyclohexane (Sigma, H4500), mefenamic acid (Sigma, M4267), aspirin (Sigma, A2093), isoniazid (Fluka, I3377), phenylbutazone (Sigma, P8386), chlorphenamine or chlorpheniramine (Sigma, C3025), amoxicillin (Sigma, 10039), 17β-estradiol (Sigma, E8875), N-nitrosodimethylamine (NDMA) (sigma, N0756), sodium hydrogen arsenate heptahydrate (Sigma, A6756), and 2,3,7,8-Tetrachlorodibenzo-p-dioxin (TCDD) (Sigma, 48599).

### Chemical Exposure to Zebrafish Fry

Homozygous LiPan fish were used to cross with wild type fish in order to obtain 100% semizygous transgenic embryos for chemical exposure experiments. Embryos were collected and incubated in egg water (60 mg/L sea salt, Red Sea) at 28°C. At ∼3 hpf, the well developing and healthy embryos were selected for chemical exposure, which was carried out in 6-well plates from 3 hpf to 120 hpf. In each well, 50 embryos were placed with 10 ml of chemical solution. Each concentration was tested in parallel in different wells with four independent replicates. The appropriate concentrations were determined by preliminary experiments with reference to previous publications, if available. Selected chemical treatment studies in zebrafish and their references are summarized in Table S1. Most of the highest concentrations used for these chemicals were below LC50 except that lindane and mefenamic acid at their highest concentration caused mortalities slightly higher than 50% (Table S2). Under these tested concentrations, there was no obvious effect on liver development as RFP-labeled livers develop around the same time in the treated fry as in the untreated controls. During the test, egg water with fresh chemicals solutions were replenished every day.

### Phenotypical Observation

During the treatment, several lethal and sublethal endpoints based on the DarT protocol [19], including survival rates, hatching rate and edema were observed and recorded as indicators for chemical toxicity. These data are presented in Table S2 and there is generally no significant abnormality observed in the low range of concentrations in these experiments.

### Imaging and Data Analysis

GFP/RFP fluorescence was observed under a fluorescent microscope (ZEISS Axiovert 200 M) with GFP/RFP filters and photographed with a digital camera (ZEISS AxiocCam HRC). For direct comparison in the same set of experiment, images were taken for the same exposure time with a fixed aperture. At least 8–10 embryos/larvae were randomly selected from each dosage group for photographing. Swimming larvae were anaesthetized with 0.1% 2-phenoxyethanol prior to photography. For each fry, under the RFP dark view, the same lateral view of the liver region of each fry was photographed with three images: one with auto exposure-time for the clearest view, one with fixed exposure-time to capture RFP intensity below saturation for intensity measurement and comparison, and the last one with sufficient exposure-time to show the whole liver region for size measurement. ImageJ software was then used to quantify RFP fluorescence intensity and liver size as we previously reported [20]. Average RFP intensity in the liver was calculated and compared within the same group of lateral view fry under the same magnification and fixed exposure-time.

For each chemical concentration, there were four replicates and each replicate had 50 embryos. Thus, 200 embryos per chemical per dose were used. The number of embryos for each lethal or sublethal endpoint was recorded and all values were computed based on the original embryo number (n = 200). P-value was calculated by t-test among the four replicates in comparison to respective controls. P<0.01 was considered highly significant difference and P<0.05 significant difference from control.

### Quantitative Real Time RT-PCR

Total RNA were extracted by Trizol based RNA extraction methods and reverse-transcribed to cDNA using Transcriptor First Strand cDNA Synthesis Kit (Roche). Quantitative PCR was carried out with LightCycler 480 SYBR Green I Master-Kit (Roche) following the MIQE (Minimum Information for Publication of Quantitative Real-Time PCR Experiments) guidelines [21]. Quantitative analyses were based on Ct values from both treated group and control group, including both target genes and housekeeping gene (β-actin). The relative expression ratio (fold change) was calculated based on ΔΔCt, ΔΔCt = (C_t,target_ −C_t,β-actin_)_treatment_ −(C_t,target_ −C_t,actin_)_control_, and fold change = 2^−ΔΔCt^.

### Cell Proliferation Assays

5-dpf zebrafish larvae were fixed in 4% paraformaldehyde in phosphate buffered saline overnight, embedded in Tissue-Tek O.C.T. compound (Sakura, Japan) for cryo-section at 5 µm thickness. PCNA antibody (AnaSpec, Singapore) together with the EnVision+System-HRP (DAB) kit (DakoCytomation, Denmark) was used to visualize proliferating cells and DAPI (4′-6-Diamidino-2-phenylindole) was used to counter-stain nuclei.

## Results

### Reduction of Liver Red Fluorescence and Size in LiPan Transgenic Fry by Hepatotoxins

**Figure 1:**
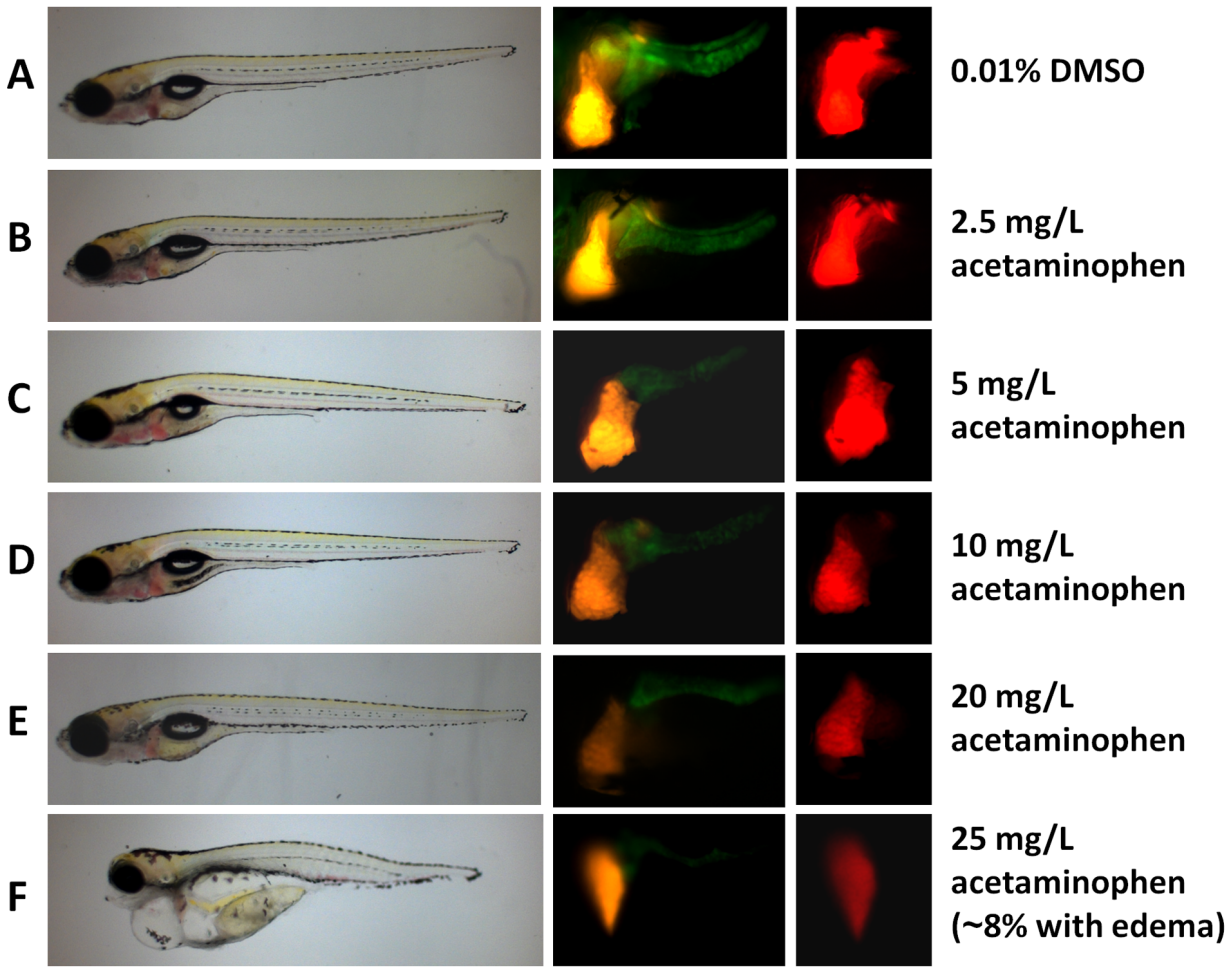
General phenotype and RFP and GFP expression in LiPan fry treated with example hepatotoxin acetaminophen. Representative fry from each treatment group with different concentrations are shown. (A) Vehicle control group with 0.01% DMSO. (B-F) Acetaminophen treatment groups with increasing concentrations of acetaminophen as indicated on the right. The left row represents general phenotype, the middle row shows liver-specific RFP and exocrine pancreas-specific GFP expression through a GFP filter (470 nm wave length), and the right row is liver-specific RFP expression through an RFP filter (546 nm wave length). Note that edema in (F) was observed only in less than 8% of 25 mg/L acetaminophen-treated fry.

**Figure 2:**
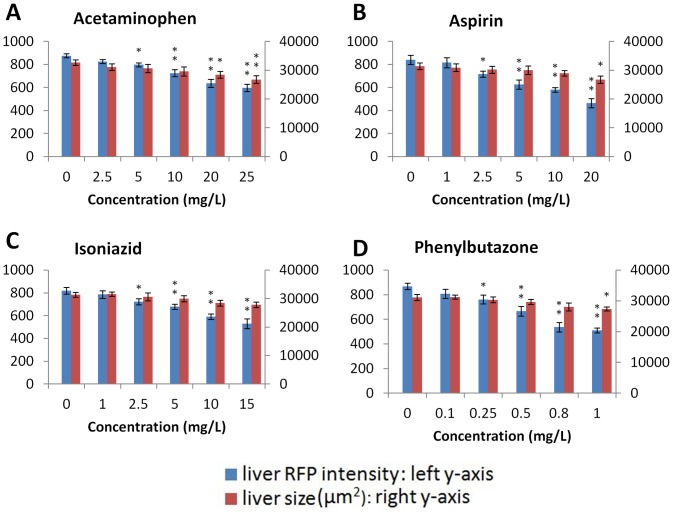
Quantitative analyses of liver RFP intensity and liver size in LiPan fry treated with four established hepatotoxins. The four hepatotoxins used, acetaminophen, aspirin, isoniazid and phenylbutazone, are indicated at the top of each panel. At least eight larvae were analysed in each concentration group. Blue bars indicate liver RFP intensity and red bars refer liver size. Asterisks indicate statistical significance: *, P<0.05; **. P<0.01.

To demonstrate the feasibility of using the LiPan transgenic fry for hepatotoxin assay, four well established hepatotoxins (acetaminophen, aspirin, isoniazid and phenylbutazone) were first tested. As exampled in Figure 1, when LiPan embryos/fry were treated with 2.5 to 25 mg/L acetaminophen from 3 hpf to 120 hpf, there was a dosage-dependent reduction of red fluorescent intensity compared to the control group with 0.01% DMSO. There was no obvious developmental abnormality up to 10 mg/L. While at the highest dose (25 mg/L), nearly 8% of surviving embryos/fry showed cardiac edema (Figure 1F), Quantitative analyses of red fluorescence intensity confirmed the dosage-dependent reduction (Figure 2A, Table S2) and statistically significant reduction (8.9%) was observed at the concentration of 5 mg/L and 32% reduction at 25 mg/L. 2D measurement of liver size as covered by the red fluorescence in the LiPan fry were also carried out and there was a similar reduction with the increase of acetaminophen concentrations (Figure 2A), and statistically significant reduction (13.0% and 18.0%) of liver size was observed at the concentrations of 20 and 25 mg/L, respectively. The GFP labeled exocrine pancreas also showed similar reduction in fluorescent intensity and the organ size (Figure 1), but we mainly focused on hepatotoxicity in the present study.

Similarly, LiPan embryos/fry were also treated with the three other hepatotoxins with a range of concentrations: 1–20 mg/L aspirin, 1–15 mg/L isoniazid and 0.1–1 mg/L phenylbutazone. As shown in Figure 2B–D and Figure S1A–C, obvious dosage-dependent reduction of liver RFP intensity was observed in all of the three hypototoxin-treated groups with the statistical significance was determined at ≥2.5 mg/L aspirin, ≥2.5 mg/L isoniazid and ≥0.25 mg/L phenylbutazone. The maximal reduction of RFP fluorescence observed were 44.8%, 35.5% and 41.1% by 20 mg/L aspirin, 15 mg/L isoniazid and 1 mg/L phenylbutazone respectively, which were higher than the maximal reduction observed from the highest acetaminophen concentration group. However, the change of liver size was not as obvious as the reduction of RFP intensity by these three hepatotoxins although there was a clear trend of reduction from all of the three groups. Statistically significant reduction was observed only from the highest concentration treatments for aspirin (20 mg/L) and phenylbutazone (1 mg/L) and the reduction was 15.9% and 12.1% respectively; however, there was no significant reduction of liver size measured from any concentration groups with the isoniazid treatment. Thus, it appears that RFP intensity is a more sensitive indicator of hepatotoxicity.

### Hepatotoxicity Tests for Chemicals Selected from Different Catagories

To further evaluate the feasibility of using the LiPan embryos/fry to develop a convenient hepatotoxicity assay, nine other chemicals of different categories were also selected for the LiPan zebrafish test. These chemicals include some well recognized environmental toxicants (lindane, arsenate, 17β-estradiol, TCDD and NDMA), some widely used human drugs (mefenamic acid, chlorphenamine, and amoxicillin), and ethanol that is known to cause liver toxicity and damages at high concentration and extended exposure [22]. Although the hepatotoxic effects have been reported for some of these chemicals such as mefenamic acid, lindane and arsenate [23]–[28], generally their potential hepatotoxicity is not well documented.

**Figure 3:**
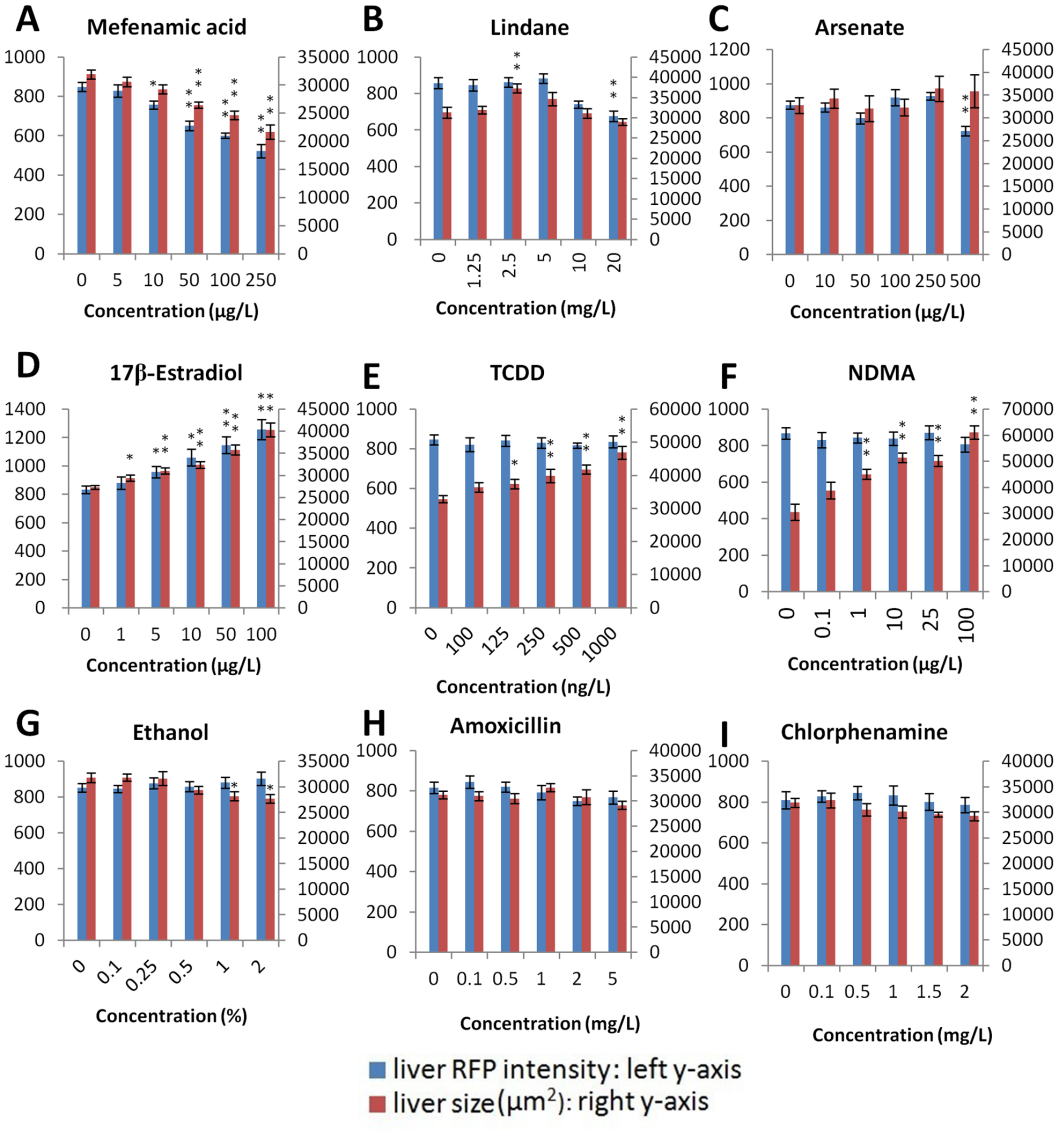
Quantitative analyses of liver RFP intensity and liver size in LiPan fry treated with nine chemicals. The names of the chemicals are indicated at the top of each panel. At least eight larvae were analyzed in each concentration group. Blue bars indicate liver RFP intensity and red bars refer liver size. Asterisks indicate statistical significance: *, P<0.05; **. P<0.01.

As shown in Figure 3A and Figure S1D, mefenamic acid was the only chemical to cause a clear dosage-dependent reduction of liver RFP fluorescence and liver size. A maximal reduction of RFP fluorescence intensity of 38.4% was observed in the highest concentration group (250 µg/L), which is comparable to the highest reduction observed from the four hepatotoxin treatments shown in Figure 2. The reduction of liver size (up to 32.1%) was much more profound than those observed from the tests with the four hepatotoxins. Thus, based on the current assay, mefenamic acid was an apparent hepatotoxin.

Other chemicals causing significant reduction of RFP fluorescence intensity in the liver included lindane (Figure 3B, Figure S1E) and arsenate (Figure 3C, Figure S1F). However, the response with lindane was not strictly dosage-dependent and arsenate caused reduction (17.5%) was observed only in the highest concentration group (500 µg/L) (Table S2).

The rest six chemicals did not cause observable reduction of RFP intensity in the liver. Instead there was an obvious dosage-dependent increase of RFP intensity in the liver by 17β-estradiol treatment, from 5.6% to 50.9% between 1 and 100 µg/L 17β-estradiol; correspondingly there was a dosage dependent increase of liver size from 7.5% to 47.6% (Figure 3D, Figure S1G, Table S2). The observation was consistent with the role of estrogen in promoting cell proliferation [29] and our observation also suggested a role of estrogen in enhancement of haptic activity. Similarly, both TCDD and NDMA treatments also showed increase of the liver size (Figure 3E and 3F, Figure S1H and S1I, Table S2), consistent with their role in promoting cell proliferation as both chemicals are considered to be carcinogens. The fact of lack of change of RFP intensity indicated that the two chemicals had no direct effects on haptic activity at the early stage of exposure. To confirm that there was an increased cell proliferation after treatments by 17β-estradiol, TCDD or NDMA. PCNA staining was performed on larvae treated by these three chemicals. Indeed there was an apparent increase of proliferating cells after treatment (Figure S2).

Ethanol treatment showed small but statistically significant reductions (11.3% and 12.8%) of the liver size at the high concentrations (1% and 2%) (Figure 3G, Figure S1J, Table S2). Combining the observation of the lack of change of RFP intensity by ethanol, these observations suggest that the adverse effect of ethanol on liver function was not apparent in an acute exposure.

The rest two chemicals, amoxicillin and chlorphenamine did not cause statistically significant change of either the RFP intensity or the liver size (Figure 3H and 3I, Figure S1K and S1L, Table S2), suggesting that their adverse role in liver function was not apparent. Consistent with this, their primary roles are not on the liver as amoxicillin is an antibiotic and chlorphenamine is a pain killer.

### Corresponding Changes of Endogenous *fabp10a* and other Lipid Pathway Genes in Response to Hepatotoxic Chemicals

**Figure 4:**
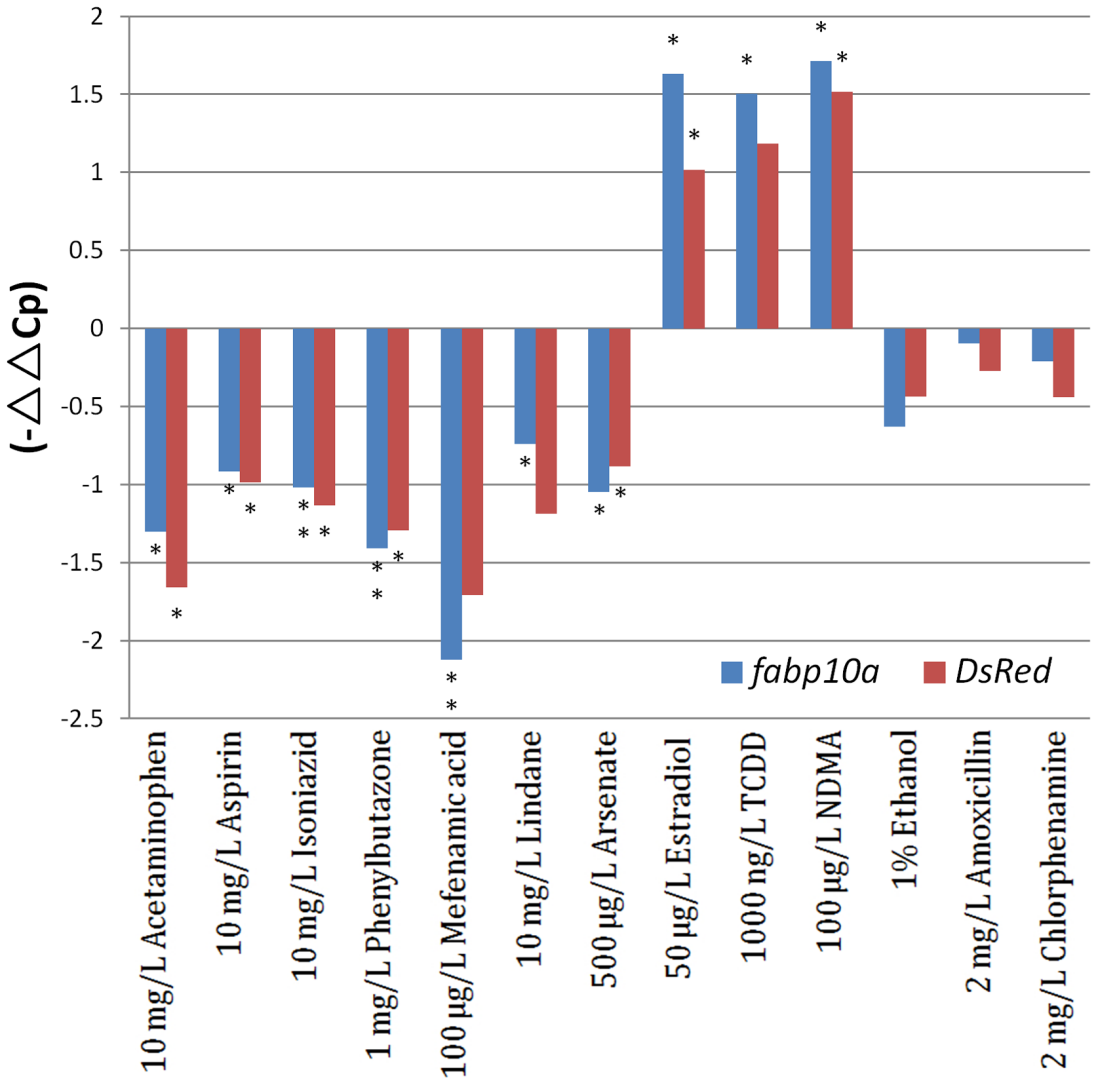
Comparison of the mRNA levels of endogenous *fabp10a* and transgenic DsRed in LiPan fry treated with variable chemicals. Quantification was carried out by RT-qPCR. Bule bars represent *fabp10a* mRNA and red bars refer *DsRed* mRNA.

In the LiPan transgenic zebrafish, RFP expression is driven by the liver specific promoter *fabp10a* [14] and thus the reduction of RFP intensity in response to hepatotoxins may reflect down-regulation of endogenous *fabp10a* gene which encode a fatty acid binding protein with functions in uptake and utilization of fatty acids as well as intracellular fatty acid transportation and metabolism [30]. To confirm the down-regulation of *fabp10a* by hepatotoxic chemicals, both endogenous *fabp10a* and *DsRed* mRNAs were measured by RT-qPCR from whole fry samples treated with 10 mg/L acetaminophen, 10 mg/L aspirin, 10 mg/L isoniazid, 1 mg/L phenylbutazone, 100 µg/L mefenamic acid, 10 mg/L lindane, 500 µg/L arsenate, 50 µg/L 17β-estradiol, 1000 ng/L TCDD, 100 µg/L NDMA, 1% ethanol, 2 mg/L amoxicillin, and 2 mg/L chlorphenamine. As shown in Figure 4, consistent with the observation on changes of DsRed fluorescence in the livers as shown in Figures 1–3, both *fabp10a* and *DsRed* mRNAs showed significant reduction in the four hepatotoxin treatment groups: acetaminophen, aspirin, isoniazid and phenylbutazone. Similarly, reduction of both *fabp10a* and *DsRed* mRNAs were also observed in the high concentration groups of mefenamic acid, lindane and arsenate treatment groups, where significant reduction of RFP fluorescence was observed (Figure 3B and 3C). As positive and negative controls, both *fabp10a* and *DsRed* mRNAs were greatly increased in the estradiol-, TCDD- and NDMA-treated groups while there was no significant change of the two mRNAs in the last three treatment groups (ethanol, amoxicillin and chlorphenamine), again consistent with the data of RFP fluorescence in the liver in the two treatment groups (Figure 3D and 3H). Thus, the change of RFP fluorescence we observed indeed reflects the level of endogenous *fabp10a* mRNA and likely the level of Fabp10a protein as well.

**Figure 5:**
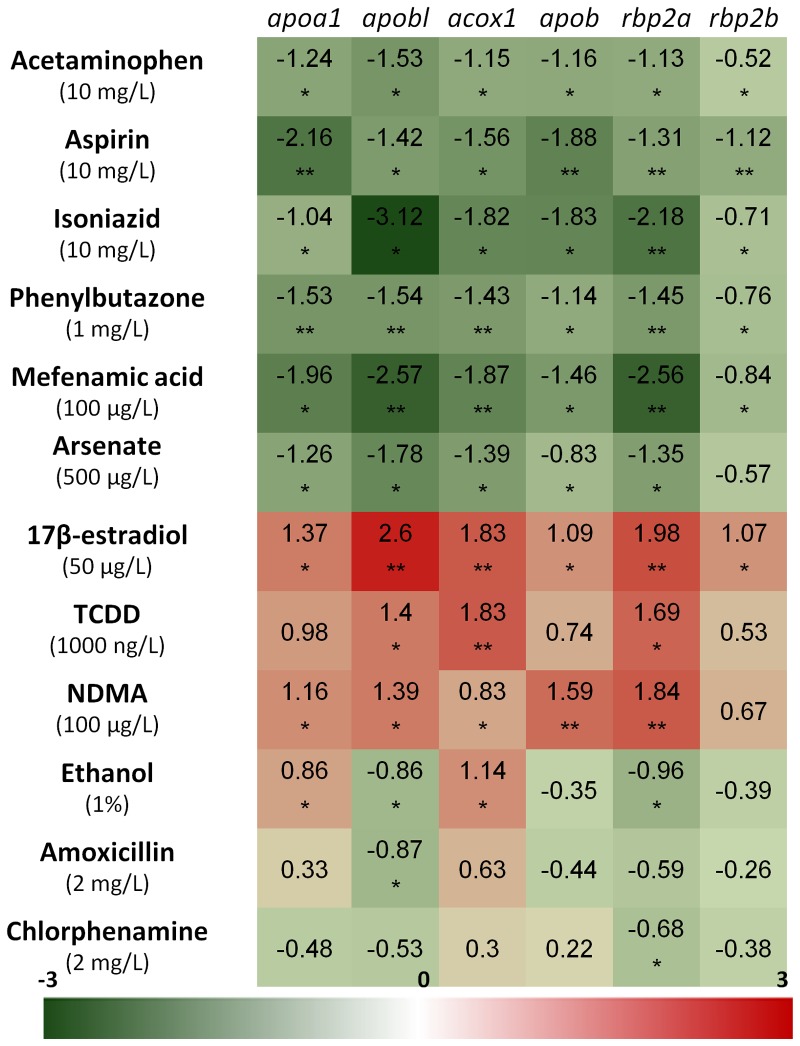
Expression of four lipid pathway genes (*appa1, apobl, acox1, apob*) and twp liver-specific genes (*rbp2a, rbp2b*) after chemical treatments. Chemical treatments were conducted from 3-qPCR was used to determine the level of gene expression. Numbers in the table are ΔΔCt value by using *β-actin* mRNA as internal control. Expression levels are represented by color gradients as indicated by the scale bar below, with the red color representing up-regulation and green color down-regulation. Gene names are indicated at the top and chemicals and their concentration used on the left. Statistical values: *, P<0.05; **. P<0.01.

To examine whether there is a general effect on lipid pathway by hepatotoxins, four lipid pathway genes were selected for RT-qPCR measurements: *apoa1* (apolipoprotein A-I), *apobl* (apolipoprotein B like), acox1 (acyl-coenzyme A oxidase 1, palmitoyl) and *apob* (apolipoprotein B). As shown in Figure 5, the expression of all of these four lipid pathway genes was significantly down-regulated by these proven hepatotoxins: acetaminophen, aspirin, isoniazid, phenylbutazone, mefenamic acid, lindane and arsenate, while their expression was up-regulated by these chemicals causing increase of either the RFP intensity or the liver size or both: 17β-estradiol, TCDD and NDMA. Similarly, another two liver specific genes, *rbp2a* (retinol binding protein 2a) and *rbp2b* (retinol binding protein 2b) were also similarly down- and up-regulated by these chemicals. Thus our quantitative gene expression assay demonstrated that there was a general connection of hepatotoxicity and lipid transportation and metabolism.

### Detection of Compound Hepatotoxic Effect from Hepatotoxin Mixture

**Figure 6:**
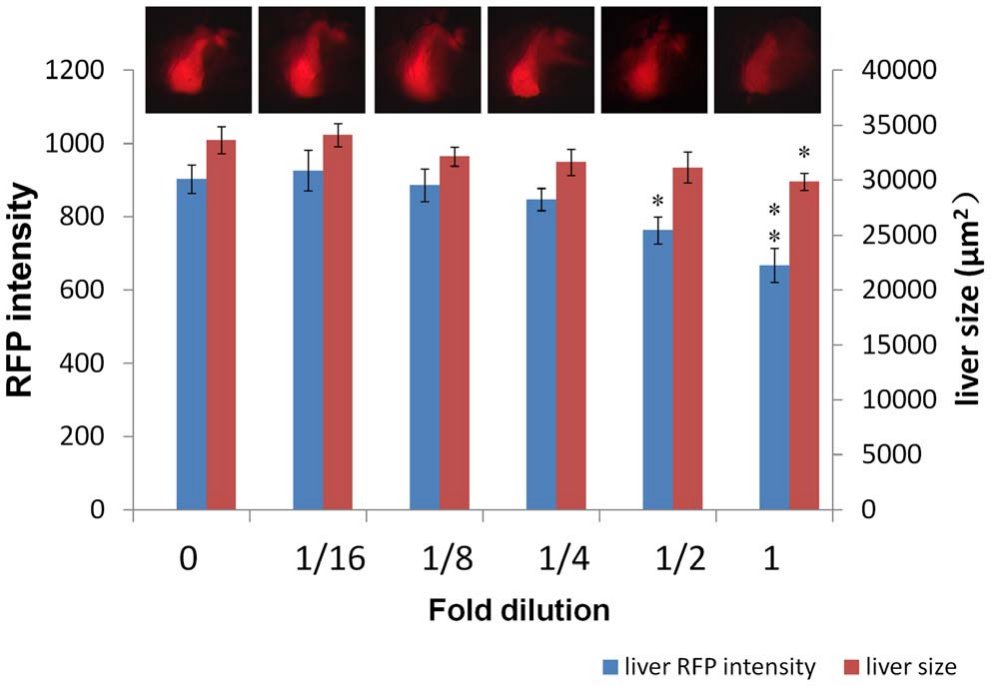
Combined hepatotoxin effect. LiPan fry were treated with a mixture of four hepatotoxins with increasing dilution factors. “1″ indicates undiluted mixture with the following final concentrations in the treatment: 2.5 mg/L acetaminophen, 1 mg/L aspirin, 1 mg/L isoniazid and 0.1 mg/L phenylbutazone. “0″ represents vehicle control groups with 0.01% DMSO. All other groups are dilutions from ½ to 1/16. Blue bars indicate liver RFP intensity and red bars refer liver size. Representative liver-specific RFP expression is shown at top of each group. Asterisks indicate statistical significance: *, P<0.05; **. P<0.01.

One important advantage of bioassay is the feasibility of analyzing biological effect from combined chemical mixture despite of undetectable levels of individual chemicals by chemical methods. To demonstrate the feasibility of using our hepatotoxicity assay to test compound effect from a mixture of chemicals, the four control hepatotoxins (acetaminophen, aspirin, isoniazid and phenylbutazone) were mixed and used in exposure experiments. We first tested the mixture of four hepatotoxins at final concentrations just below their effective concentrations: 2.5 mg/L acetaminophen, 1 mg/L aspirin, 1 mg/L isoniazid and 0.1 mg/L phenylbutazone, at which concentrations none of the hapatotoxins alone led to significant change of liver RFP intensity and liver size (Figure 2). As shown in Figure 6 and Table S2, a highly significant 26.1% reduction of liver fluorescence intensity, accompanied with a significant 11.2% reduction of liver size, was observed. Interestingly, when the test was carried out at a half of the concentration, a significant reduction (15.5%) of liver fluorescence intensity was still observed (Figure 6, Table S2). In general, there was a dosage-dependent decrease of the hepatotoxic effect when the mixture was further diluted but the changes of both liver fluorescence intensity and liver size were not statistically significant after four fold dilution compared to those in the 0.01 DMSO control group.

## Discussion

Based on an extensive analysis of hepatic transcriptomic data obtained from zebrafish treated with various environmental chemicals, we have previously reported the existence of an inverted profile of two hub biological pathways in response to chemical perturbations [31]. One set was primarily associated with lipid metabolism while the other set was non-lipid pathways. Up-regulation of several lipid-related pathways are generally accompanied with down-regulation of the other set of non-lipid pathways, or vice versa [31]. Since change in lipid metabolism is often an important feature during chemical perturbation, expression of *fabp10a,* which codes for a liver fatty acid binding protein, is likely a valid marker of chemical-induced hepatotoxicity as reflected by liver RFP intensity in LiPan transgenic zebrafish. This observation was further supported by RT-qPCR measurement of the expression of four other lipd pathway related genes (Figure 5). For chemicals that exert toxicity via a lipid independent mechanism, RFP fluorescence in LiPan fry also facilitates the observation and measurement of the liver size which may be altered when there is a severe effect on liver or when the chemicals induce hypertrophy or atrophy.

In the present study, we first demonstrated with four well established hepatotoxins that both liver RFP intensity and the liver size in LiPan fry were significantly reduced by the four hepatotoxins (acetaminophen, aspirin, isoniazid and phenylbutazone). Previously, it has also been reported the reduction of GFP fluorescent intensity by treatment with acetaminophen in another transgenic zebrafish line, *Tg(fabp10:GFP)* [32], indicating the robustness of the fluorescence based assay for hepatotoxicity. As negative controls, neither chlorphenamine nor amoxicillin significantly alter RFP intensity and liver size in LiPan fry even at concentrations that increased edema rate or reduced hatching rate. This is not surprising since both drugs are not considered to be hepatotoxins. Chlorphenamine is an antihistamine and is used to relieve allergic conditions [33] while amoxicillin is an antibiotics although it may cause cholestasis when it is co-administered with clavulanate [34]. In contrast, the rest seven chemicals tested caused the changes of either liver RFP intensity or liver size, or both. Some of these drugs have been previously reported to incur liver injury while there is no information for many others regarding their potential hepatotoxicity.

In addition to the four positive control hepatotoxins (acetaminophen, aspirin, isoniazid and phenylbutazone), mefenamic acid also caused decrease of both liver RFP intensity and liver size. Aspirin, phenylbutazone and mefenamic acid are non-steroidal anti-inflammatory drugs (NSAIDs), a major class of analgesics characterized by their inhibitory effects on cyclooxygenase (COX) enzymes and the second most common cause of drug induced liver injury [35], [36]. The COX enzyme system converts arachidonic acid to cyclic endoperoxide prostaglandin G2, which is further processed by other enzymes to form various prostaglandins, mediators of pain transmission. Mechanisms of hepatotoxicity of NSAIDs are not yet clear and they may be class-specific but COX-2 inhibition has been shown to be associated with hepatotoxicity. [36], [37]. To date, 21 NSAIDs in the market have been reported to cause chronic liver injury or acute idiosyncratic liver damages, among which aspirin accounts for 12% of the total cases [38]. Phenylbutazone was introduced in 1949 to treat rheumatoid arthritis but removed from the market in many countries since 1983 due to its toxicity to multiple organs including liver. It is now mainly used in horses with reduced dosage. Although the hepatotoxicity of phenylbutazone is well documented, its mechanism is poorly understood, partly due to the lack of studies after its withdrawal [39], [40]. Mefenamic acid is not generally considered as a hepatotoxin, as there has been only one published case of severe but non-fatal hepatic necrosis [41]. A 14-day study in mice, with a relatively high dose of 50 mg/kg/day and 100 mg/kg/day, suggested that mefenamic acid could lead to hepatocellular necrosis, degeneration and inflammation [42]. Our current assay demonstrated the potential of mefenamic acid causing liver damage within 5 days in larvae. The relation between FABP (fatty acid binding protein) abundance and COX enzyme inhibition has not been reported; the reduction of RFP intensity observed in these three NSAID treated groups could be a result of either direct perturbation of fatty acids metabolism pathway or a secondary effect of server liver damage with reduced liver size.

In addition, both lindane and arsenate treatments showed clearly the reduction of liver RFP intensity at high concentrations used in the present study but the change in the liver size was not significant. Arsenate has been found to alter lipid metabolism [43], [44] and to reduce the mRNA of a adipocyte fatty acid binding protein (aP2) during *in vitro* adipogenesis [45], consistent with our current findings. Interestingly, in an adult zebrafish acute exposure set-up, fatty acid binding proteins were found to be up-regulated by arsenate in male fish but not female fish. [44], [46]. It may suggest that the effect of arsenate on lipid metabolism is sex-dependent; however, our embryo assay evaluates toxicity without discrimination of sexes. Lindane, a persistent organochlorinated pesticide with main adverse effects in nervous system, has been reported to cause liver injury and fatty infiltration to liver [47]–[49]. The toxicity is believed to be at least partly due to induction of oxidative stress [48], [49]. However, there are no clear associations between lindane and fatty acid binding proteins, except that several studies have indicated association between lindane intake and altered lipid composition [48], [49], lipogenic enzymes [50] or lipid peroxidation [51]. In contrast, ethanol is known to cause fatty liver toxicity [22] but we did not detect a significant change of liver RFP intensity in the acute exposure and its effect on the liver could be more prominent on chronic exposure. Nevertheless, we observed that acute ethanol treatment resulted in marked reduction of liver size when the treatment concentration was 1% or higher.

Estrogens and functional estrogen receptors have been shown to have protective effect against high fat-diet induced obesity and fatty liver in a few animal studies [52]–[54]. It is thus interesting to note that estradiol treatment led to significant increase of both liver RFP intensity and liver size, which may indicate enhancement of liver function. Similarly, both TCDD and NDMA also caused liver enlargement but not an increase of liver RFP intensity. Our PCNA staining assays confirmed the enhanced cell proliferation activity in the liver by these chemical treatments. Both TCDD and NDMA are classified as carcinogens by IARC (The International Agency for Research on Cancer). TCDD is an IARC Group 1 carcinogen that has been shown to promote liver tumour formation in various animal models [55]. TCDD has been shown to cause heptatomegaly in rats and mice [56]–[58]. NDMA is a “probable carcinogen” (Group 2A) as classified by IARC and it is found to be positively associated with cancer formation in various animal models [59], including the zebrafish [60]. Hepatomegaly was also observed in early animal studies of acute NDMA toxicity and also some human exposure cases [61], [62]. While TCDD caused significant liver enlargement only at a relatively high concentration of 125 ng/L with accompanying reduction in hatching rate, NDMA affected liver size from 1 µg/L onwards while there was no noticeable changes in gross appearance based on conventional toxicological endpoints such as DarT [19].

Serum LFABP (liver fatty acid binding protein) has been used as a biomarker for screening acute kidney damage for years [63]. Decreased LFABP has been found to be a hepatocellular adenoma marker specific to a subtype *HNF1α* mutation [64], similar to our earlier observation in carcinogen-induced liver tumors in zebrafish [65], [66]. Here we reported that both *lfabp10a* (or *lfabp*) mRNA and RFP intensity in the LiPan fry sensitively reflected chemical-induced liver toxicity. As shown in Figures 3 and 4, most changes in RFP intensity and liver size are within one fold, while changes in the level of *fabp10a* mRNA were generally greater than two fold. The smaller fold change at protein level than its mRNA level is commonly observed when protein and RNA data are compared [67]–[69]. The greater fold change at the transcript level indicates that mRNA quantification is a more sensitive measurement due to its greater range of detection. Nonetheless, direct visualization of RFP fluorescence is convenient and can be performed in live specimen repeatedly for multiple time-points. Furthermore, RFP fluorescence also reveals liver morphological change that is not possibly available from mRNA measurement. Current data on liver size is based on 2D imaging; if 3D visualization of liver RFP is performed, the sensitivity of detection is expected to be higher. However, 3D images and analyses are time- and cost-consuming, thus not suitable for rapid analyses. Another advantage of the current assay is the feasibility to develop into a high throughput screening assay using imaging systems and computer algorithms that are already available [70], [71]. For example, Kanungo et al have reported an automated high content screening method for in vivo measurement of axon length of zebrafish fry; the assay was carried out in a 384-well plate format and fluorescent labels in axons were automatically captured and quantitatively analyzed [72]. Similarly, each LiPan embryo can be placed in individual well for simultaneous imaging and computerized analysis in high throughput setting.

In a critical review using data from ten biggest drug companies, it has been estimated that from 1991 to 2000 only 11% of drugs that entered clinical trials were successfully brought to the market, and the two top causes of failure were the lack of efficacy and safety. One of the main underlying causes of such high attrition rate is believed to be a lack of reliable predictive preclinical toxicity studies, particularly in *in vivo* animal models [73]. Currently, toxicity assay is heavily dependent on in vitro cell-based assays, but there are major limitations in the cell-based toxicity assays, including lack of organ-specific toxicity mechanisms and limited use of pre-lethal indicators [74]. While more cell-based *in vitro* assays have been developed in the past decade, development of *in vivo* models has much lagged behind. Rodent models such as mice, rat, guinea pigs and rabbit are often used and provide useful toxicology data, yet the high maintenance cost as well as long study time required make them undesirable for large scale or high throughput studies. Besides, those models may either have relatively low occurrence of liver injury or require very stringent exposure conditions that may not be constant to human [75]. Thus, the LiPan transgenic zebrafish may provide a convenient and useful tool for rapid *in vivo* screening of chemicals for potential hepatotoxicity to supplement current hepatotoxicity assays.

## Supporting Information

Figure S1 Liver-specific RFP expression from representative fry treated with various chemicals at different concentrations. (A-L) Treatments with different chemicals as indicated on the right. Each row represents each chemical and concentrations are indicated within each panel. 0 indicates control groups with 0.01% DMSO except for the controls for arsenate and ethanol groups where the controls were egg water.
(PDF)

Figure S2 Increased hepatocyte proliferation induced by 17β-estradiol (B), TCDD (C) and NDMA (D). Zebrafish larvae were treated 50 µg/L 17β-estradiol. 1000 ng/L TCDD or 100 µg/L NDMA from 3 dpf to 5 dpf and liver cryo-sections were prepared and stained by DAPI and PCNA. Increased numbers of proliferating hepatocytes were noted from 17β-estradiol. TCDD and NDMA treatment groups compared to the 0.01% DMSO vehicle control group.
(PDF)

Table S1 Selected chemical treatment studies in zebrafish.
(PDF)

Table S2 Summary of survival rates, hatching rates, edema rates, liver RFP intensities and liver sizes in LiPan fry treated with various chemicals at different concentrations. Statistical analyses were carried out by t-test between treated and control groups: red highlights represent P<0.01 and yellow highlight 0.05.
(PDF)
