# Supplementary material for: Development of a Convenient In Vivo Hepatotoxin Assay Using a Transgenic Zebrafish Line with Liver-Specific DsRed Expression
Source: PLoS One. 2014 Mar 13;9(3):e91874. doi: 10.1371/journal.pone.0091874 (PMC3953600; doi:10.1371/journal.pone.0091874)
Supplement: Figure S1 — Liver-specific RFP expression from representative fry treated with various chemicals at different concentrations. (A-L) Treatments with different chemicals as indicated on the right. Each row represents each chemical and concentrations are indicated within each panel. 0 indicates control groups with 0.01% DMSO except for the controls for arsenate and ethanol groups where the controls were egg water. (PDF) [file pone.0091874.s001.pdf]

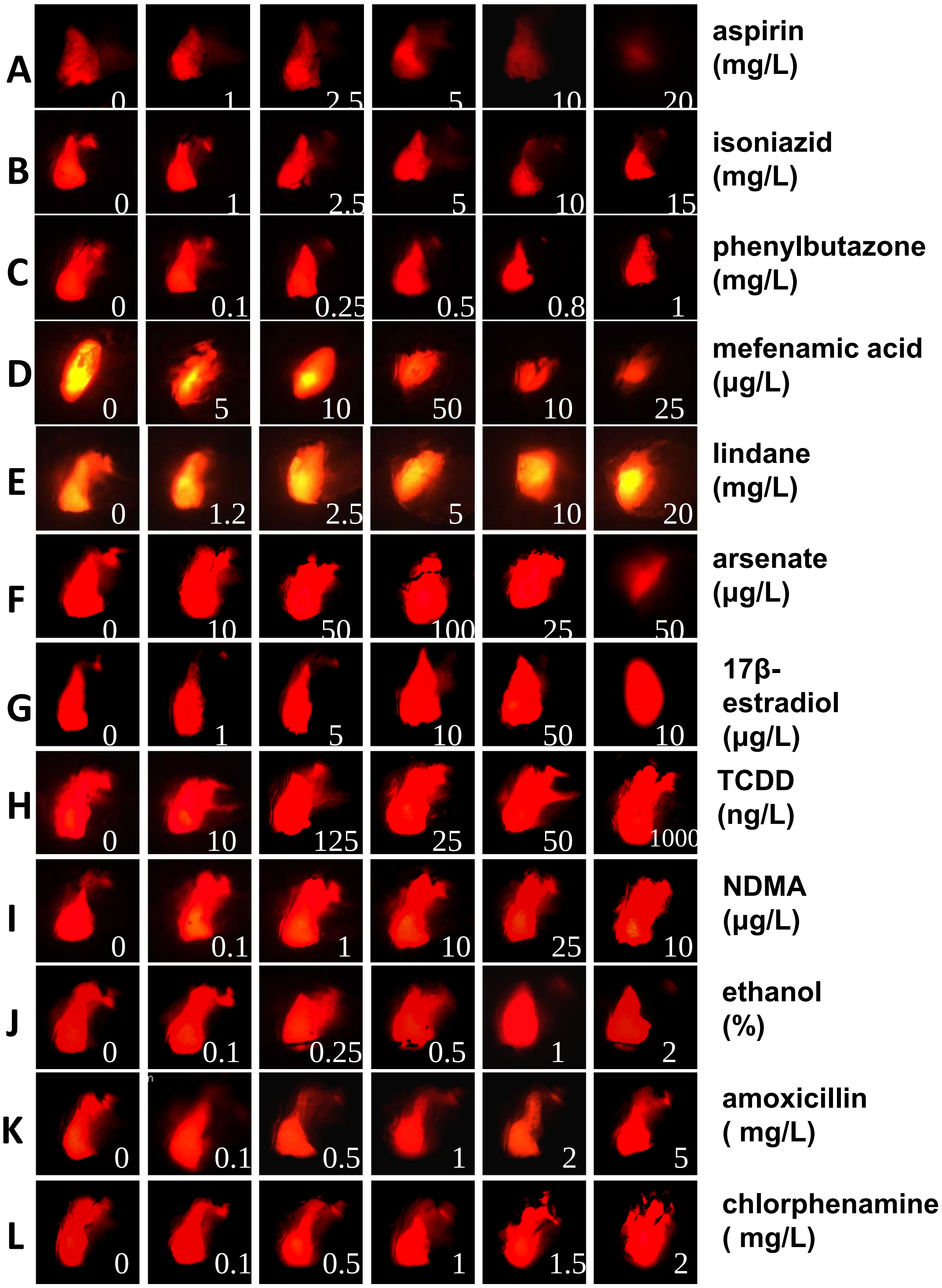

Figure S1. Liver-specific RFP expression from representative fry treated with various chemicals at different concentrations. (A-L) Treatments with different chemicals as indicated on the right. Each row represents each chemical and concentrations are indicated within each panel. 0 indicates control groups with 0.01% DMSO except for the controls for arsenate and ethanol groups where the controls were egg water.
