# Supplementary material for: Development of a Convenient In Vivo Hepatotoxin Assay Using a Transgenic Zebrafish Line with Liver-Specific DsRed Expression
Source: PLoS One. 2014 Mar 13;9(3):e91874. doi: 10.1371/journal.pone.0091874 (PMC3953600; doi:10.1371/journal.pone.0091874)
Supplement: Figure S2 — Increased hepatocyte proliferation induced by 17β-estradiol (B), TCDD (C) and NDMA (D). Zebrafish larvae were treated 50 µg/L 17β-estradiol. 1000 ng/L TCDD or 100 µg/L NDMA from 3 dpf to 5 dpf and liver cryo-sections were prepared and stained by DAPI and PCNA. Increased numbers of proliferating hepatocytes were noted from 17β-estradiol. TCDD and NDMA treatment groups compared to the 0.01% DMSO vehicle control group. (PDF) [file pone.0091874.s002.pdf]

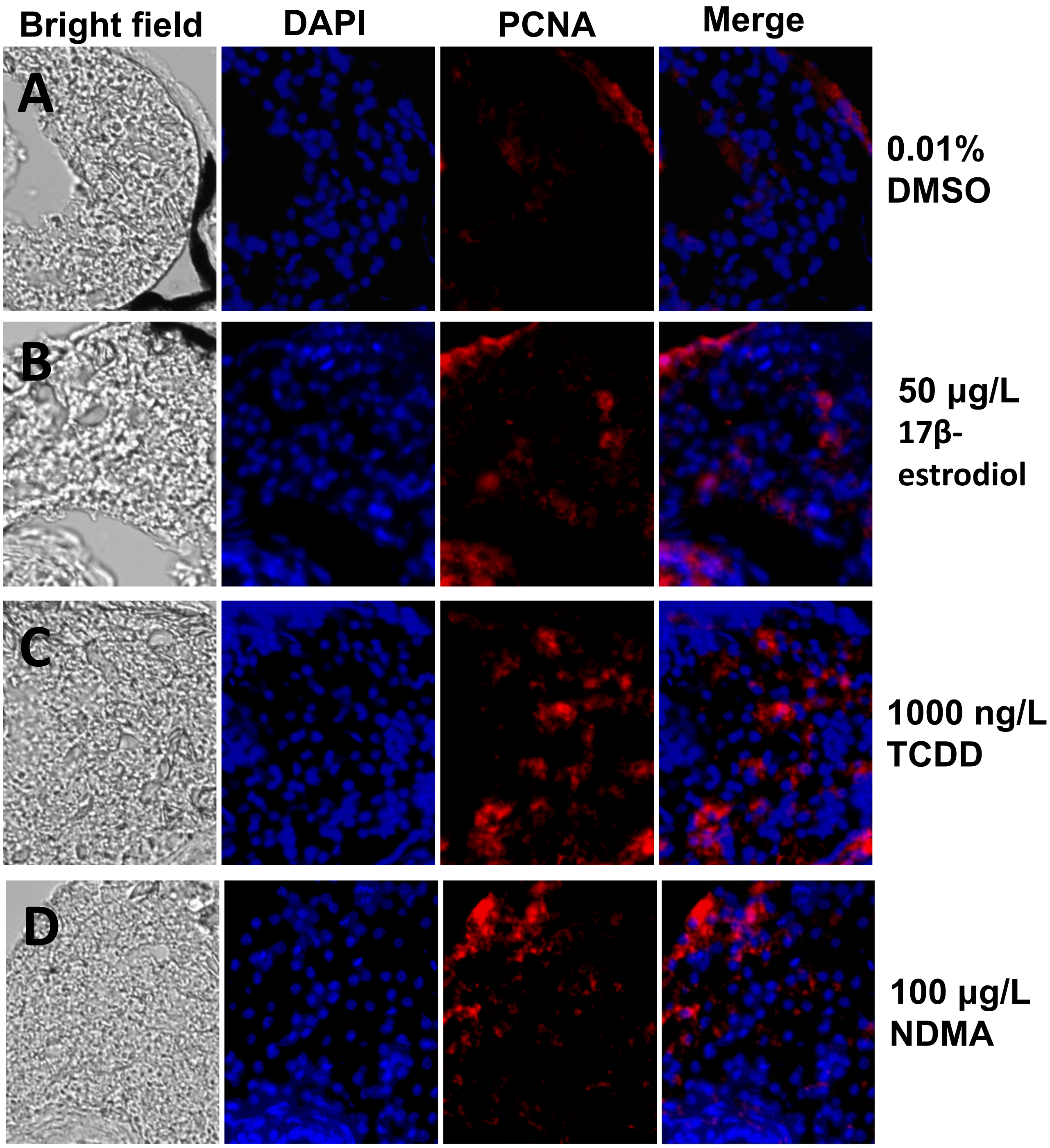


**Figure S2. Increased hepatocyte proliferation induced by 17β-estradiol (B), TCDD (C) and NDMA (D).** Zebrafish larvae were treated 50 µg/L 17β-estradiol. 1000 ng/L TCDD or 100 µg/L NDMA from 3 dpf to 5 dpf and liver cryo-sections were prepared and stained by DAPI and PCNA. Increased numbers of proliferating hepatocytes were noted from 17β-estradiol. TCDD and NDMA treatment groups compared to the 0.01% DMSO vehicle control group.
