# Supplementary material for: Development of a Convenient In Vivo Hepatotoxin Assay Using a Transgenic Zebrafish Line with Liver-Specific DsRed Expression
Source: PLoS One. 2014 Mar 13;9(3):e91874. doi: 10.1371/journal.pone.0091874 (PMC3953600; doi:10.1371/journal.pone.0091874)
Supplement: Table S1 — Selected chemical treatment studies in zebrafish. (PDF) [file pone.0091874.s003.pdf]

Table S1. Selected chemical treatment studies in zebrafish

Chemicals	Concentrations
Acetaminophen	0.1-1000 μM (0.015-151.16 mg/L) in zebrafish larva [1]<br>2- 6 mM (302.32-906.96 mg/L) in zebrafish embryos [2]<br>5-10 mM (755.81-1511.63 mg/L) in zebrafish embryos / adult [3]
Aspirin	0.1- 1000 μM (0.018-180.16mg/L) in zebrafish larva [1]<br>200 mg/L in zebrafish embryos [4]
Isoniazid	20–320 μg/L in zebrafish embryos [5]
Phenylbutazone	No zebrafish data available.
Mefenamic acid	10-60 μM (2.41-14.46 mg/L) in zebrafish adult[6]<br>0.25-17.16 mg/L in zebrafish embryos [7]
Lindane	40-150 μg/L in zebrafish embryos [8]<br>80 μg/L in zebrafish embryos [9]
Arsenate	0.05-15 mg /L in zebrdish adult[10],<br>LC50: 56 mg/L in zebrdish adult [11]
Estradiol	0.05-10 ng /L in zebrafish embryos [12]<br>5 -100 ng/L in zebrdish adult [13]
TCDD	0.1-10 nM(32.20-3219.7 ng/L) in zebrdish embryos / adult [14] [15]
NDMA	50 mg/L in 8-week-old zebrafish [16]
Ethanol	1.5% in zebrdish adult [17]<br>0.1-2% in zebrdish adult [18]
Amoxicillin	75-1125 mg/L, in zebrafish embryos, 48h premature hatching<br>EC50=132.4 mg/L [19]
Chlorphenamine	No zebrafish data available.

References:

1. He JH, Guo SY, Zhu F, Zhu JJ, Chen YX, et al. (2013) A zebrafish phenotypic assay for assessing drug-induced hepatotoxicity. J Pharmacol Toxicol Methods 67: 25-32.
2. Weigt S, Huebler N, Braunbeck T, von Landenberg F, Broschard TH (2010) Zebrafish teratogenicity test with metabolic activation (mDarT): effects of phase I activation of acetaminophen on zebrafish Danio rerio embryos. Toxicology 275: 36-49.
3. North TE, Babu IR, Vedder LM, Lord AM, Wishnok JS, et al. (2010) PGE2-regulated wnt signaling and N-acetylcysteine are synergistically hepatoprotective in zebrafish acetaminophen injury. Proceedings of the National Academy of Sciences of the United States of America 107: 17315-17320.
4. Praskova E, Zivna D, Stepanova S, Sevcikova M, Blahova J, et al. (2012) Acute toxicity of acetylsalicylic acid to juvenile and embryonic stages of Danio rerio. Neuro Endocrinol

Lett 33 Suppl 3: 72-76.
5. Strecker R, Weigt S, Braunbeck T (2013) Cartilage and bone malformations in the head of zebrafish (Danio rerio) embryos following exposure to disulfiram and acetic acid hydrazide. Toxicol Appl Pharmacol 268: 221-231.
6. Nadanaciva S, Aleo MD, Strock CJ, Stedman DB, Wang H, et al. (2013) Toxicity assessments of nonsteroidal anti-inflammatory drugs in isolated mitochondria, rat hepatocytes, and zebrafish show good concordance across chemical classes. Toxicol Appl Pharmacol 272: 272-280.
7. Collard HR, Ji K, Lee S, Liu X, Kang S, et al. (2013) Toxicity and endocrine disruption in zebrafish (Danio rerio) and two freshwater invertebrates (Daphnia magna and Moina macrocopa) after chronic exposure to mefenamic acid. Ecotoxicol Environ Saf 94: 80-86.
8. Gorge G, Nagel R (1990) Toxicity of lindane, atrazine, and deltamethrin to early life stages of zebrafish (Brachydanio rerio). Ecotoxicol Environ Saf 20: 246-255.
9. Ensenbach U, Nagel R (1995) Toxicity of complex chemical mixtures: acute and long-term effects on different life stages of zebrafish (Brachydanio rerio). Ecotoxicol Environ Saf 30: 151-157.
10. Baldissarelli LA, Capiotti KM, Bogo MR, Ghisleni G, Bonan CD (2012) Arsenic alters behavioral parameters and brain ectonucleotidases activities in zebrafish (Danio rerio). Comp Biochem Physiol C Toxicol Pharmacol 155: 566-572.
11. Liu F, Gentles A, Theodorakis CW (2008) Arsenate and perchlorate toxicity, growth effects, and thyroid histopathology in hypothyroid zebrafish Danio rerio. Chemosphere 71: 1369-1376.
12. Segner H, Navas JM, Schafers C, Wenzel A (2003) Potencies of estrogenic compounds in in vitro screening assays and in life cycle tests with zebrafish in vivo. Ecotoxicol Environ Saf 54: 315-322.
13. Versonnen BJ, Arijs K, Verslycke T, Lema W, Janssen CR (2003) In vitro and in vivo estrogenicity and toxicity of o-, m-, and p-dichlorobenzene. Environ Toxicol Chem 22: 329-335.
14. Li ZH, Xu H, Zheng W, Lam SH, Gong Z (2013) RNA-Sequencing Analysis of TCDD-Induced Responses in Zebrafish Liver Reveals High Relatedness to In Vivo Mammalian Models and Conserved Biological Pathways. PLoS One 8: e77292.
15. Ton C, Lin Y, Willett C (2006) Zebrafish as a model for developmental neurotoxicity testing. Birth Defects Res A Clin Mol Teratol 76: 553-567.
16. Mizgireuv IV, Majorova IG, Gorodinskaya VM, Khudoley VV, Revskoy SY (2004) Carcinogenic effect of N-nitrosodimethylamine on diploid and triploid zebrafish (Danio rerio). Toxicol Pathol 32: 514-518.
17. Kashyap B, Pegorsch L, Frey RA, Sun C, Shelden EA, et al. (2013) Eye-Specific Gene Expression following Embryonic Ethanol Exposure in Zebrafish: Roles for Heat Shock Factor 1. Reprod Toxicol.
18. Chatterjee D, Shams S, Gerlai R (2014) Chronic and acute alcohol administration induced neurochemical changes in the brain: comparison of distinct zebrafish populations. Amino Acids.
19. Oliveira R, McDonough S, Ladewig JC, Soares AM, Nogueira AJ, et al. (2013) Effects of oxytetracycline and amoxicillin on development and biomarkers activities of zebrafish (Danio rerio). Environ Toxicol Pharmacol 36: 903-912.
