# Supplementary material for: Development of a Convenient In Vivo Hepatotoxin Assay Using a Transgenic Zebrafish Line with Liver-Specific DsRed Expression
Source: PLoS One. 2014 Mar 13;9(3):e91874. doi: 10.1371/journal.pone.0091874 (PMC3953600; doi:10.1371/journal.pone.0091874)
Supplement: Table S2 — Summary of survival rates, hatching rates, edema rates, liver RFP intensities and liver sizes in LiPan fry treated with various chemicals at different concentrations. Statistical analyses were carried out by t-test between treated and control groups: red highlights represent P<0.01 and yellow highlight 0.05. (PDF) [file pone.0091874.s004.pdf]

Table S2. Summary of survival rates, hatching rates, lack of edema, liver RFP intensities and liver sizes in LiPan fry treated with various chemicals at different concentrations. Statistical analyses were carried out by t-test between treated and control groups: red highlights represent P<0.01 and yellow highlight 0.05.

Chemicals	Concentration		Survival rate	Hatching	Lack of edema	Liver RFP intensity (% change)	2D liver size (μm^2) (% change)
0.01% DMSO	0	mg/L	85.5%	85.5%	85.0%	876 (0)	32674 (0)
Acetaminophen	2.5	mg/L	84.5%	84.5%	84.5%	826 (-5.7)	31090 (-4.8)
	5	mg/L	82.5%	81.5%	81.0%	798 (-8.9)	30647 (-6.2)
	10	mg/L	71.0%	68.5%	67.5%	725 (-17.2)	29622 (-9.3)
	20	mg/L	64.0%	57.0%	59.5%	636 (-27.4)	28441 (-13)
	25	mg/L	53.0%	46.5%	48.5%	596 (-32.0)	26793 (-18)
0.01% DMSO	0	mg/L	86.0%	86.0%	85.5%	840 (0)	31398 (0)
Aspirin	1	mg/L	84.5%	84.0%	84.5%	816 (-2.9)	30922 (-1.5)
	2.5	mg/L	81.5%	79.0%	80.5%	715 (-14.9)	30198 (-3.8)
	5	mg/L	76.0%	74.5%	74.0%	625 (-25.6)	30026 (-4.4)
	10	mg/L	70.5%	68.0%	66.5%	581 (-30.8)	28929 (-7.9)
	20	mg/L	58.5%	53.0%	54.5%	464 (-44.8)	26718 (-14.9)
0.01% DMSO	0	mg/L	85.5%	85.5%	85.0%	820 (0)	31391 (0)
Isoniazid	1	mg/L	81.5%	80.5%	81.0%	786 (-4.1)	31597 (0.7)
	2.5	mg/L	76.0%	74.5%	75.0%	723 (-11.8)	30624 (-2.4)
	5	mg/L	70.5%	68.5%	67.0%	678 (-17.3)	29940 (-4.6)
	10	mg/L	64.5%	61.0%	59.0%	591 (-27.9)	28437 (-9.4)
	15	mg/L	57.5%	52.5%	51.0%	529 (-35.5)	27830 (-11.3)
0.01% DMSO	0	μg/L	84.5%	83.5%	84.0%	868 (0)	31122 (0)
Phenylbutazone	0.1	mg/L	82.0%	81.0%	81.5%	808 (-6.9)	31241 (0.4)
	0.25	mg/L	78.5%	77.5%	77.0%	762 (-12.2)	30357 (-2.5)
	0.5	mg/L	74.5%	73.0%	72.5%	667 (-23.2)	29668 (-4.7)

	0.8	mg/L	65.5%	61.0%	62.0%	537 (-38.1)	28027 (-9.9)
	1	mg/L	53.0%	49.0%	50.5%	511 (-41.1)	27368 (-12.1)
0.01% DMSO	0	µg/L	87.5%	87.0%	87.5%	848 (0)	31931 (0)
Mefenamic acid	5	µg/L	81.0%	81.0%	79.5%	827 (-2.5)	30613 (-4.1)
	10	µg/L	75.5%	74.0%	74.5%	757 (-10.7)	29267 (-8.3)
	50	µg/L	71.0%	68.5%	66.0%	651 (-23.2)	26458 (-17.1)
	100	µg/L	57.5%	49.0%	51.0%	600 (-29.2)	24671 (-22.7)
	250	µg/L	40.5%	34.5%	35.5%	522 (-38.4)	21670 (-32.1)
0.01% DMSO	0	mg/L	83.5%	83.5%	83.0%	857 (0)	31293 (0)
Lindane	1.25	mg/L	74.5%	72.5%	72.0%	847 (-1.2)	31942 (2.1)
	2.5	mg/L	68.5%	64.5%	63.5%	862 (0.6)	37284 (19.1)
	5	mg/L	56.5%	53.5%	55.5%	883 (3)	34671 (10.8)
	10	mg/L	48.0%	46.0%	46.5%	740 (-13.7)	31076 (-0.7)
	20	mg/L	33.5%	26.5%	29.0%	676 (-21.1)	28950 (-7.5)
egg water	0	µg/L	86.0%	86.0%	86.0%	875 (0)	32755 (0)
Arsenate	10	µg/L	83.5%	83.0%	82.0%	862 (-1.5)	34257 (4.6)
	50	µg/L	79.0%	74.0%	74.5%	798 (-8.8)	32079 (-2.1)
	100	µg/L	70.5%	67.0%	66.0%	920 (5.1)	32322 (-1.3)
	250	µg/L	63.0%	58.5%	56.5%	928 (6.1)	36434 (11.2)
	500	µg/L	59.5%	51.5%	53.0%	722 (-17.5)	35863 (9.5)
0.01% DMSO	0	µg/L	84.5%	84.5%	84.0%	833 (0)	27328 (0)
17β-estradiol	1	µg/L	85.0%	84.0%	84.5%	880 (5.6)	29384 (7.5)
	5	µg/L	83.0%	83.0%	82.0%	957 (14.9)	31014 (13.5)
	10	µg/L	81.5%	81.0%	81.5%	1060 (27.3)	32395 (18.5)
	50	µg/L	79.0%	77.0%	77.5%	1148 (37.8)	35800 (31)
	100	µg/L	76.5%	75.0%	73.5%	1257 (50.9)	40348 (47.6)
0.01% DMSO	0	ng/L	85.5%	85.5%	85.0%	846 (0)	32851 (0)
0.01% Toluene	0	ng/L	85.0%	84.5%	84.5%	837(-1.1)	31972(-2.7)
TCDD	100	ng/L	84.5%	84.5%	84.5%	821 (-3)	36381 (10.7)

	125	ng/L	81.5%	80.5%	81.0%	842 (-0.5)	37419 (13.9)
	250	ng/L	76.0%	73.5%	74.5%	830 (-1.9)	39867 (21.4)
	500	ng/L	71.5%	69.0%	67.5%	817 (-3.4)	41796 (27.2)
	1000	ng/L	64.0%	59.5%	60.5%	836 (-1.2)	46898 (42.8)
0.01% DMSO	0	µg/L	86.5%	86.5%	86.5%	868 (0)	30473 (0)
NDMA	0.1	µg/L	87.5%	87.0%	87.0%	831 (-4.3)	38841 (27.5)
	1	µg/L	84.5%	84.5%	84.5%	843 (-2.9)	45055 (47.9)
	10	µg/L	82.0%	80.5%	81.5%	838 (-3.5)	51418 (68.7)
	25	µg/L	81.0%	78.5%	79.0%	872 (0.5)	50211 (64.8)
	100	µg/L	75.5%	74.5%	74.0%	807 (-7)	61059 (100.4)
egg water	0	%	85.5%	85.0%	85.5%	853 (0)	31777 (0)
Ethanol	0.1	%	84.5%	84.5%	83.5%	846 (-0.8)	31846 (0.2)
	0.25	%	81.0%	80.0%	79.0%	878 (2.9)	31661 (-0.4)
	0.5	%	77.5%	74.0%	73.0%	859 (0.7)	29370 (-7.6)
	1	%	66.0%	57.5%	58.5%	882 (3.4)	28189 (-11.3)
	2	%	51.5%	40.5%	42.0%	903 (5.9)	27725 (-12.8)
0.01% DMSO	0	mg/L	84.5%	84.0%	84.5%	815 (0)	31205 (0)
Amoxicillin	0.1	mg/L	83.0%	83.0%	83.0%	844 (3.6)	30988 (-0.7)
	0.5	mg/L	79.0%	76.5%	77.5%	820 (0.6)	30485 (-2.3)
	1	mg/L	72.0%	69.0%	71.0%	793 (-2.7)	32678 (4.7)
	2	mg/L	65.5%	60.5%	57.5%	749 (-8.1)	30772 (-1.4)
	5	mg/L	52.5%	49.0%	47.5%	768 (-5.8)	29164 (-6.5)
0.01% DMSO	0	mg/L	85.5%	85.5%	85.5%	811 (0)	31892 (0)
Chlorphenamine	0.1	mg/L	82.5%	82.5%	81.5%	829 (2.2)	32370 (1.5)
	0.5	mg/L	78.5%	78.0%	76.5%	845 (4.2)	30495 (-4.4)
	1	mg/L	74.0%	72.5%	69.5%	834 (2.8)	30131 (-5.5)
	1.5	mg/L	63.5%	60.5%	59.0%	801 (-1.2)	29611 (-7.2)
	2	mg/L	56.0%	51.5%	52.5%	787 (-3)	29285 (-8.2)
0.01% DMSO	0		85.0%	84.5.0%	84.5%	903 (0)	33646 (0)

Hepatotoxin mixture*	1/16	85.5%	84.5.0%	84.0%	927 (2.6)	34114 (1.4)
	1/8	84.5%	83.0%	83.5%	886 (-1.9)	32150 (-4.4)
	1/4	81.0%	79%	79.5%	848 (-6.1)	31625 (-6.0)
	1/2	79.5%	74.0%	73.5%	763 (-15.5)	31149 (-7.4)
	1	74.5%	71.0%	71.5%	667(-26.1)	29863 (-11.2)

* 1X hepatotoxins mixture contains 2.5 mg/L acetaminophen, 1 mg/L aspirin, 1 mg/L isoniazid and 0.1 mg/L phenylbutazone.
